# Preclinical studies of NOS inhibitor T1059 vasopressor activity on the models of acute hemorrhagic shock in rats and dogs

**DOI:** 10.3389/fphar.2022.995272

**Published:** 2022-09-30

**Authors:** Marina Filimonova, Ljudmila Shevchenko, Victoria Makarchuk, Alina Saburova, Petr Shegay, Andrey Kaprin, Sergey Ivanov, Alexander Filimonov

**Affiliations:** ^1^ A. Tsyb Medical Radiological Research Center—Branch of the National Medical Research Radiological Center of the Ministry of Health of the Russian Federation, Obninsk, Russia; ^2^ National Medical Research Radiological Center of the Ministry of Health of the Russian Federation, Obninsk, Russia; ^3^ Medical Institute (RUDN University), Peoples’ Friendship University of Russia, Moscow, Russia

**Keywords:** hemorrhagic shock, *in vivo* models, vasopressor activity, NOS inhibitor, rats, dogs

## Abstract

The development of new effective and safe vasopressors is one of the ways to increase the effectiveness of the treatment of hypotensive disorders, the severe forms of which remain a common cause of death in all countries of the world. Previously, we synthesized the original compound T1059, a selective inhibitor of eNOS/iNOS which has a pronounced vasoconstrictive effect. Here we show its vasopressor activity in models of the early stage of acute hemorrhagic shock in rats and dogs, as part of preclinical studies. The results indicate NOS inhibitor T1059 as a potent long-acting vasopressor. Its single parenteral administration in sufficiently safe doses (1/50–1/9 LD_10_), caused in rats and dogs a rapid increase in vascular tone, accompanied by a prolonged hypertensive effect (within 90–120 min in rats, and within 115 min in dogs). The repeated administration of T1059 at low doses (1/3 of the first dose) made it possible to considerably (by at least 60 min) prolong a significant vasopressor effect. In all schemes, T1059 administration considerably inhibited the development of threatening cardiorespiratory disorders and significantly (*p* = 0.0026–0.0098) increased the short-term survival of experimental animals, formally extending the duration of the “golden hour” by 2 times. These data indicate that NOS inhibitors and, in particular, compound T1059, are able to create new opportunities in the treatment of hypotensive disorders, including the provision of assistance at the prehospital stage of treatment of such pathologies.

## Introduction

**FIGURE 1:**
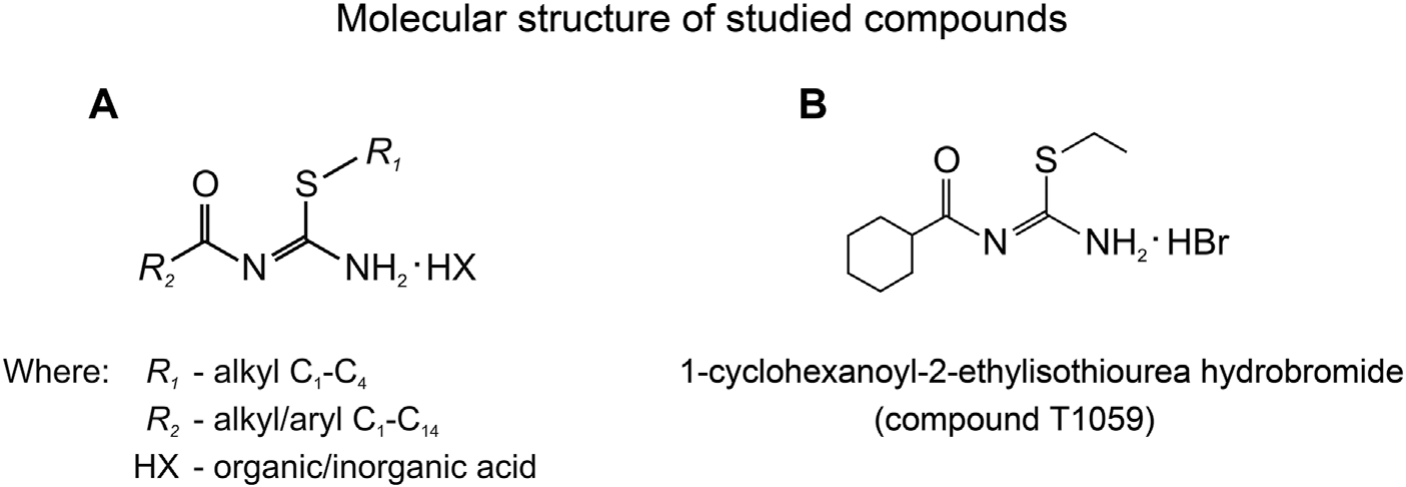
**(A)** General chemical structure of N,S-substituted isothioureas, synthesized in the A. Tsyb MRRC laboratory of radiation pharmacology, exhibiting significantly NOS-inhibiting activity. **(B)** Molecular structure of 1-cyclohexanoyl-2-ethylisothiourea hydrobromide (compound T1059).

The lab of radiation pharmacology of the A. Tsyb Medical Radiological Research Center (A. Tsyb MRRC) has long been interested in the chemistry and pharmacology of signaling pathway modifiers, including NO donors and NOS inhibitors. So, some time ago, while screening linear and cyclic isothioureas, we identified and synthesized a large group of isothiourea derivatives (Figure 1A) that are competitive inhibitors of NOS (Proskuryakov et al., 2002, 2005; Proskuryakov et al., 2010; Filimonova et al., 2012a). Further, we found that some compounds of this group (such as T1023, T1082, T1084) are promising for further pharmacological development—as hypoxic radioprotectors that provide effective prevention of hematopoietic and gastrointestinal acute radiation syndromes, and radiotherapy complications (Filimonova et al., 2020b, 2021, 2022b; Saburova et al., 2020), as well as antitumor, antiangiogenic agents (Filimonova et al., 2019a; 2019b; 2022a).

Along with this, one of the studied compounds, T1059, 1-cyclohexanoyl-2-ethylisothiourea hydrobromide (Figure 1B), demonstrated a high affinity for eNOS and iNOS, as well as a pronounced vasotropic activity. In preliminary studies in rats, compound T1059 had a long-term vasoconstrictive effect at a single parenteral administration (i.p, i.v. and i.m.) at relatively safe doses (5–30 mg/kg; 1/50–1/10 LD_10_) (Filimonova et al., 2018). 2–5 min after T1059 administration and the next 70–140 min, an increase in peripheral vascular resistance (by 30%–60%) in rats was recorded. Later on, all changes in these animals normalized independently as the vascular tone weakened. In rats with various hypovolemic disorders (on models of acute hemorrhagic shock and acute endotoxemic shock), T1059 at such doses and methods of administration realized the same long-term vasoconstrictor effect (Filimonova et al., 2020a). Under these conditions, T1059-induced vasoconstriction did not cause a significant baroreflex response in rats. On these models, T1059 effect was accompanied by a pronounced, stable hypertensive effect. It is duration significantly (7–10 times) exceeded the duration of the effects of high doses of phenylephrine with the same methods of administration.

These experimental data justified the feasibility of conducting preclinical studies of pharmacological activity and safety of T1059 as a vasopressor agent (state contract with the Ministry of Education and Science of the Russian Federation #14.N08.11.0078). The vascular and hemodynamic effects of T1059 were studied in models of hypotensive disorders of various etiology and course in small and large laboratory animals—acute hemorrhagic shock and acute endotoxemic shock in rats and dogs, gangliolytic hypotension and refractory endotoxemic vasoplegia in rats. A vast amount of data has been obtained that needs a detailed presentation. In this publication, we present the first part of the experimental data—studies on models of acute hemorrhagic hypotension in rats and dogs. We also plan to present the results obtained on models of vasodilatory disorders in a subsequent publication.

## Materials and methods

### Animals

Male Wistar rats (3–4 months old, 180–260 g) and male and female outbreed dogs (2–4 years old, 9–19 kg) were used in these studies. Rats were purchased from the Biomedical Technology Scientific Center of Federal Biomedical Agency of Russia. Rats were housed in the vivarium of the A. Tsyb MRRC in T-3 cages under natural light conditions with forced ventilation 16 times per hour, at a room temperature 18–20°C and relative humidity 40–70%. Animals had free access to the filtered (Aquaphor, Russia) water and feed for rodents PK-120–1 (Laboratorsnab, Russia). Outbred dogs were received from the sponsor according to the Donation Agreement. Dogs were kept in single aviaries in the A. Tsyb MRRC vivarium under similar conditions of lighting, temperature and air ventilation. The dogs were fed twice a day. The dogs’ diet included Now Natural Holistic adult dog food (Petcurean, Canada), raw meat, boiled fish, meat-based soups with cereals and vegetables. Animal studies were approved by the A. Tsyb MRRC Ethical Committee, were performed in accordance with generally accepted standards for the animal treatment, based on standard operating procedures of the A. Tsyb MRRC, in accordance with the rules and requirements of the European Convention ETS/STE No 123 and international standard GLP (OECD Guide 1:1998).

### T1059: Synthesis, toxicological and biochemical properties, administration

Method of T1059 synthesis was developed in the laboratory of radiation pharmacology A. Tsyb MRRC (Filimonova et al., 2015, 2018). It represents the reaction between 1-cyclohexanoylthiourea and the excess of bromethyl in an inert organic solvent at elevated temperature. Synthesis example: a mixture of 1-cyclohexanoylthiourea (3.7 g, 20 mmol), ethyl bromide (4.4 g, 40 mmol) and dry acetonitrite (10 ml) in a sealed ampoule was heated in a boiling water bath for 20 h, the residue was filtered off and recrystallized twice from 4-methyl-2-pentanone to yield a crystalline T1059 (2.8 g, 48.5%). These methods ensured a stable quality of a substance with a content of 1-cyclohexanoyl-2-ethylisothiourea hydrobromide of more than 95% and a total content of related and extraneous impurities of less than 0.5% of dry weight. Compound T1059 is a white crystalline powder substance that is easily soluble in water, acetone and chloroform, and insoluble in hexane. Its molecular weight is 295.2; spectra ^1^H PMR (500 MHz, DMSO-d6, δ): 1.3 (m, 9H); 1.62–1.84 (m, 5H); 3.65 (m, 1H); 10.8 (b, 3H). Melting temperature is 123–125°C. In the benzene-ethanol-triethylamine system 9:1:0.1 the Rf value is 0.35. Its 1% aqueous solution is transparent and colorless with pH = 3.92 at 20°C.

Compound T1059 belong to the “moderately hazardous” class (Berezovskaya, 2003; Gad, 2007). Its acute toxicity parameters LD_10_, LD_16_, LD_50_ and LD_84_ estimated by the Litchfield-Wilcoxon method (Mironov, 2012) are 260, 278, 310 and 362 mg/kg i. p, respectively, for Wistar rats. With i.g. administration, the sensitivity of rats to T1059 toxic effect is reduced by 5–7 times, so in this case its LD_10_, LD_16_, LD_50,_ and LD_84_ values are 1,320, 1,440, 2030 and 3,480 mg/kg, respectively. With long-term administration, T1059 does not show cumulative toxic properties [cumulative toxicity Lim test (Lim et al., 1961)].

In radiological studies *in vitro* on isolated recombinant human NOS isoforms by the rate of accumulation of [^3^H]-L-citrulline (van Eijk et al., 2007) T1059 effectively inhibits NOS with significant selectivity (15–30 times) to endothelial and inducible isoforms—its IC_50_ values for nNOS, iNOS and eNOS are 60.1, 1.8 and 3.3 μM, respectively (Filimonova et al., 2018). The mechanism of inhibition of all NOS isoforms is competitive, fully reversible. Its NOS inhibitory activity *in vivo* is quite pronounced. According to EPR spectrometry with a diethyldithiocarbamate trap (Hogg, 2010), already in the first 30 min after T1059 single i.p. administration at doses 1.5–30 mg/kg, a significant decrease in spontaneous and lipopolysaccharide-induced NO production was observed in various tissues of mice. Increasing the dose of T1059 causes both the increase of the degree (from 55% to 97%) and the duration (from 1 to 4–5 h) of suppression of NO endogenous production (Filimonova et al., 2018).

In this study, T1059 was used as aqueous solutions for single and double parenteral (i.v. and i.m.) administration. T1059 solutions were prepared *ex tempore* based on water for injection (Dalchimpharm, Russia). In a rat model of acute hemorrhagic shock, T1059 was administered once, 5 or 10 mg/kg i.v. (1.0 ml/kg of 0.5% or 1% solutions; slowly, 0.1 ml/min); 30 mg/kg i.m. (2.0 ml/kg of 1.5% solution; slowly, 0.2 ml/min) or twice, 30 + 10 mg/kg i.m. (2.0 ml/kg + 0.66 ml/kg of 1.5% solution; slowly). The used doses of T1059 for rats were justified in preliminary studies in this model of hemorrhagic shock (Filimonova et al., 2020a). Control (untreated) rats received once i.v. 1.0 ml/kg of 0.9% sodium chloride for injections (Biochemist, Russia). In a model of acute hemorrhagic shock in dogs, T1059 was administered once, 3.2 mg/kg i.v. (0.25 ml/kg of 1.28% solution; slowly, 2.0 ml/min) or 9.5 mg/kg i.m. (0.5 ml/kg of 1.9% solution in two slow injections in both thighs). The doses of T1059 used in dogs were equivalent to those for rats, 10 mg/kg (i.v.) and 30 mg/kg (i.m.), taking into account the body surface area of laboratory animals (Mironov, 2012). Control (untreated) dogs received once i.v. or i.m. 0.25 ml/kg of 0.9% sodium chloride for injections.

### Acute hemorrhagic hypotension models, study design

The vasopressor activity of T1059 was studied in models of acute severe hemorrhagic shock in rats and acute moderate hemorrhagic shock in dogs caused by massive blood loss (BL). In rat studies, animals were anesthetized (thiopental sodium, Sandoz, Austria; 60 mg/kg, i.p.), tracheostomy was placed, jugular vein and carotid arteries catheterizations were performed, invasive blood pressure (BP) sensors and ECG electrodes were connected, and heparin were injected (Heparin sodium, Ozon, Russia; 100 ME, i.v). After stabilization of the animal’s condition, baseline ECG (standard leads) and indicators of systolic and diastolic BP (SBP and DBP), heart rate (HF) and respiratory rate (RF) were recorded using a polygraph RM-6000 (Nihon Kohden, Japan) or the PowerLab 8/30 complex (ADInstruments, Australia). To reproduce acute severe hemorrhagic shock, blood was taken from the right carotid artery for 8–12 min in a volume of 25 ml/kg. For rats, this BL corresponds to a loss of 40% of circulating blood volume (Diehl et al., 2001). At the end of blood sampling, the registration of indicators was repeated. Then the control rats received a single i.v. injection of 0.9% sodium chloride solution (1 ml/kg), and the rats of the experimental groups were given a single injection of T1059: i.v.—at doses of 5 or 10 mg/kg (1.0 ml/kg of 0.5% or 1% solutions; slowly); i.m.—at dose of 30 mg/kg (2.0 ml/kg of 1.5% solution; slowly). Further recording of ECG and physiological parameters in these animals was continued for the next 120 min. In experiments with double i. m. administration of T1059, the first injection (30 mg/kg; 2.0 ml/kg of 1.5% solution; slowly) was given immediately after BL, and the second injection (10 mg/kg; 0.66 ml/kg of 1.5% solution; slowly)—after 70–140 min, at a time when the vasopressor effect from the first injection became weakened to a level of negligible. Further monitoring in these cases was continued until 60 min after the second injection. Euthanasia of anaesthetized rats that survived to the end of monitoring was carried out by air embolism.

For studies in dogs, animals were accustomed within 1 week to the manipulation room, short-term (10–30 min) fixations, BP measurement procedures and ECG recording. One day before the study, the hair on their limbs removed (trimmer Moser ChroMini Type 1,591, Germany) to access the necessary vessels. Baseline SBP, DBP, HF and RF were measured 30 min after sedation bromdihydrochlorphenylbenzodiazepine (elzepam, Ellara, Russia; 0.1 mg/kg, i.v.). BP и HF were measured in arteria brachialis using a veterinary tonometer petMAP graphic II (CardioCommand, United States). ECG registration was performed using a cardiograph Cardiofax GEM (Nihon Kohden, Japan). Then, controlled blood sampling in the amount of 20 ml/kg from vena cephalica or vena saphenus lateral tarsal was performed for 15–20 min using Helmflon venous catheters (20 g 1.1 × 33 mm; Helm, Germany). For dogs, such BL corresponds to a loss of 25% of circulating blood volume (Diehl et al., 2001). At the end of blood sampling, complete hemostasis was ensured and ECG and physiological parameters were recorded again. Then, the control animals received a single i.v. injection of 0.9% sodium chloride solution (0.25 ml/kg), and the dogs of the experimental groups received T1059 once: i.v.—at dose 3.2 mg/kg (0.25 ml/kg of 1.28% solution; slowly); i.m.—at dose 9.5 mg/kg (0.5 ml/kg of 1.9% solution in two injections, slowly, in both thighs). Subsequent recording of ECG and physiological parameters was continued for 120 min. Further observations and daily examination of these animals were carried out for the next 15 days.

T1059 vasopressor activity was assessed by intergroup statistical comparison of hemodynamic parameters in untreated and T1059-treated animals, as well as by the quantitative severity and duration of the hypertensive effect. In addition, the effect of T1059 on the clinical course of the early stage of acute severe hemorrhagic shock in rats was indirectly assessed by the dynamics of ECG, external respiration parameters and Kaplan-Meier diagrams of short-term (2–3 h) survival.

### Statistical analysis

Standard parameters of variation statistics were calculated for all experimental data and their values are given (including graphically) as M ± SD. For multiple intergroup and intragroup comparisons of physiological parameters, the level of significance of differences was assessed using the Kruskal–Wallis ANOVA by ranks with post hoc Mann–Whitney *U* test with Bonferroni–Holm corrections for multiple comparison (Holm, 1979); and for multiple comparisons of survival diagrams, using the χ^2^ test with post hoc Cox F test with Bonferroni–Holm corrections. In all cases, effects or differences were considered statistically significant at the 5% level.

## Results

### Effects of T1059 on the model of acute severe hemorrhagic shock in rats

**FIGURE 2:**
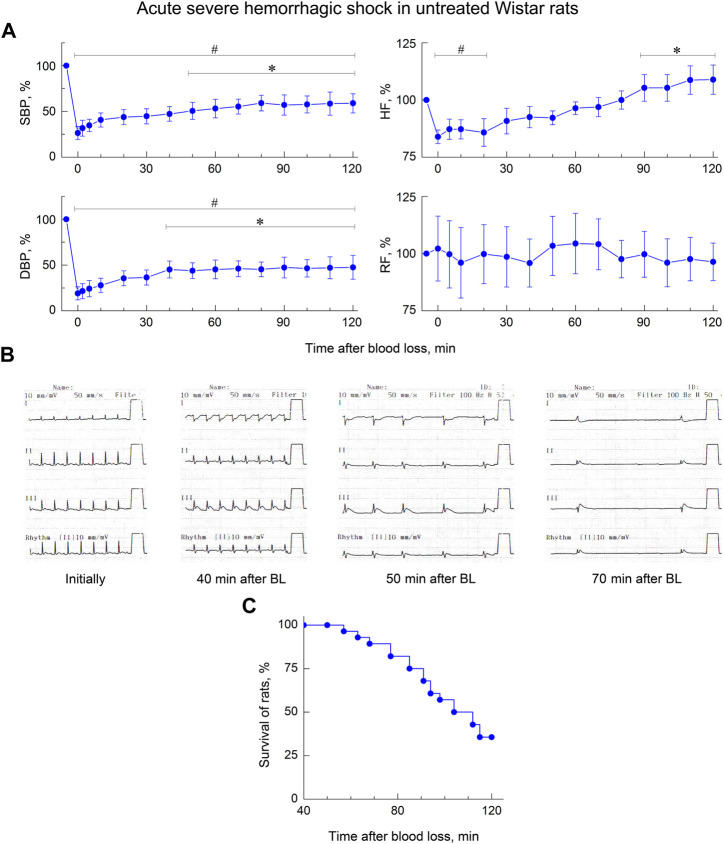
Early stage of acute severe hemorrhagic shock in untreated anesthetized Wistar rats. **(A)** Dynamics of indicators of systolic and diastolic blood pressure (SBP and DBP), heart frequency (HF) and respiratory frequency (RF). Data are normalized to the original values of indicators of animals and presented as percentage. Graphical deviations correspond to SD (n = 10–28 per point). # - significantly different vs. original value of indicator (SBP: *p* < 0.00001, *p* < 0.00001, *p* < 0.00001, *p* = 0.00002, *p* = 0.00005, *p* = 0.00007, *p* = 0.00023, *p* = 0.00049, *p* = 0.00072, *p* = 0.00148, *p* = 0.00136, *p* = 0.00219, *p* = 0.00165, *p* = 0.00374, *p* = 0.00531, respectively; DBP: *p* < 0.00001, *p* < 0.00001, *p* < 0.00001, *p* < 0.00001, *p* = 0.00003, *p* = 0.00008, *p* = 0.00027, *p* = 0.00019, *p* = 0.00076, *p* = 0.00061, *p* = 0.00052, *p* = 0.00263, *p* = 0.00175, *p* = 0.00360, *p* = 0.00574, respectively; HF: *p* = 0.00311, *p* = 0.00628, *p* = 0.00543, *p* = 0.01947, respectively). *—significantly different vs. value of indicator immediately after blood loss (SBP: *p* = 0.03810, *p* = 0.02795, *p* = 0.01436, *p* = 0.00252, *p* = 0.00469, *p* = 0.00327, *p* = 0.00708, *p* = 0.00851, respectively; DBP: *p* = 0.02462, *p* = 0.03513, *p* = 0.01745, *p* = 0.00826, *p* = 0.00504, *p* = 0.00977, *p* = 0.00758, *p* = 0.01476, *p* = 0.02635, respectively; HF: *p* = 0.02914, *p* = 0.02373, *p* = 0.00668, *p* = 0.00812, respectively). **(B)** Typical cardio-respiratory dynamics in untreated rats. Initially: BP—133/86 mmHg; HF—451 min-1; sinus rhythm, no changes in the ECG; RF—62 min-1. 40 min after blood loss (BL): BP—59/35; HF—417; sinus rhythm, ECG sings of myocardial ischemia; RF—38. 50 min after BL: BP—55/26; HF—146–206; sinus arrhythmia, sings of myocardial ischemia; terminal respiration, RF—7–12. 70 min after BL: BP—30/19; HF—57; complete atrioventricular block, myocardial ischemia; respiratory movements are not recorded. At 73 min a cardiac arrest was recorded. **(C)** Short-term survival of untreated rats (*n* = 28). Diagram was plotted using Kaplan-Meier method.

In rat studies, a simultaneous (within 8–12 min) controlled loss of 40% of circulating blood (25 ml/kg) led to the development of acute severe hemorrhagic shock in anesthetized animals. At the end of blood sampling, all experimental rats showed severe hypotension - the average values of SBP and DBP in these animals were 33 and 19 mmHg (respectively, 26% and 22% of the level before BL). The depth of hypotension in control untreated rats during the first 60–90 min after BL was partially compensated by an increase in vascular tone and acceleration of the heart rate (Figure 2A)—by this time, the mean SBP and DBP in these animals had risen to 66 and 37 mmHg (respectively, 53% and 44% of the level before BL). But such adaptation, apparently, did not significantly compensate the lack of blood flow. In most of the control rats, against the background of maintaining such a significant hypotension, manifestations of cardiorespiratory insufficiency began to increase 40–70 min after BL (Figure 2B)—total myocardial ischemia developed, atrioventricular conduction became grossly disturbed, breathing slowed down and acquired an arrhythmic, terminal character, so that cessation of respiratory movements and heart contractions was soon recorded. In this model of acute hemorrhagic shock, 18 (64%) out of 28 control rats died within 120 min of observation (Figure 2C).

**FIGURE 3:**
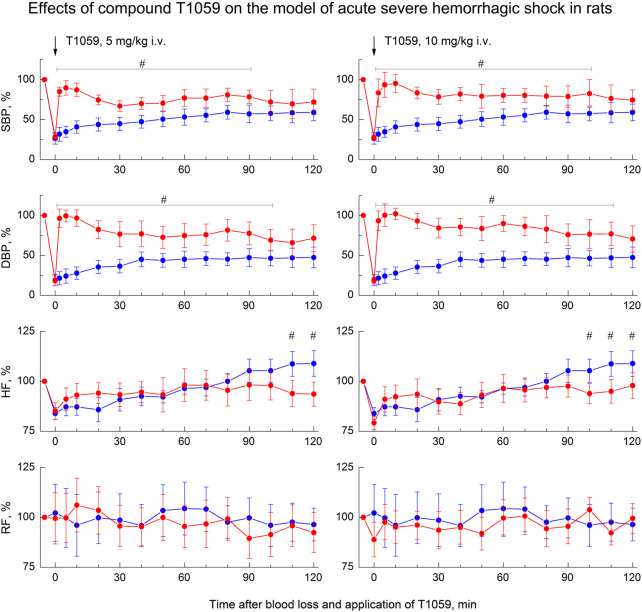
Effect of compound T1059 with a single i.v. injection at doses of 5 mg/kg (left) and 10 mg/kg (right) on hemodynamics and external respiration of Wistar rats at an early stage of acute severe hemorrhagic shock. Red symbols and lines are indicators of T1059-treated rats (*n* = 12–15 per point), blue symbols and lines are indicators of untreated rats (*n* = 10–28). Data are normalized to the original values of indicators of animals and presented as percentage. Graphical deviations correspond to SD. # - significantly different indicators in untreated vs. T1059-treated rats (5 mg/kg, SBP: *p* = 0.00002, *p* < 0.00001, *p* = 0.00004, *p* = 0.00073, *p* = 0.00462, *p* = 0.00218, *p* = 0.02964, *p* = 0.00871, *p* = 0.01053, *p* = 0.00932, *p* = 0.03817, respectively; DBP: *p* < 0.00001, *p* < 0.00001, *p* < 0.00001, *p* = 0.00006, *p* = 0.00175, *p* = 0.00322, *p* = 0.00789, *p* = 0.00648, *p* = 0.00470, *p* = 0.00091, *p* = 0.00467, *p* = 0,02306, respectively; HF: *p* = 0.03715, *p* = 0.01872, respectively. 10 mg/kg, SBP: *p* = 0.00027, *p* = 0.00008, *p* < 0.00001, *p* = 0.00036, *p* = 0.00084, *p* = 0.00053, *p* = 0.00779, *p* = 0.00092, *p* = 0.00415, *p* = 0.01930, *p* = 0.04706, *p* = 0.03021, respectively; DBP: *p* = 0.00001, *p* < 0.00001, *p* < 0.00001, *p* = 0.00005, *p* = 0.00017, *p* = 0.00063, *p* = 0.00508, *p* = 0.00024, *p* = 0.00136, *p* = 0.00790, *p* = 0.01455, *p* = 0.02643, *p* = 0.02768, respectively; HF: *p* = 0.03180).

As in preliminary studies (Filimonova et al., 2018; 2020a) on this model of hemorrhagic shock, a single parenteral administration of T1059 after BL, caused a rapid development of a pronounced and prolonged vasoconstrictive, hypertensive effect in hypotensive rats. T1059 vasopressor activity after i.v. injections at doses of 5 or 10 mg/kg (1/50 or 1/25 LD_10_) had a noticeable biphasic character (Figure 3). In the first 15–20 min, the effect was most pronounced and was achieved already by the 2^nd^ minute after the injection. During this period of time, the SBP and DBP values in rats in a state of severe hemorrhagic shock were 100–120 and 75–85 mmHg (respectively, 80%–95% and 90–100% of the level before BL). In the subsequent period of time, the hypertensive effect of T1059 in these animals was somewhat less pronounced—the average SBP and DBP were 85–95 и 60–70 mmHg (respectively, 70%–80% and 70%–85% of the level before BL). The duration of a significant vasopressor effect of T1059 with a single i.v. injection was high—90 min at a dose of 5 mg/kg and 100 min at a dose of 10 mg/kg А significant increase in DBP was observed up to 100 and 110 min.

**FIGURE 4:**
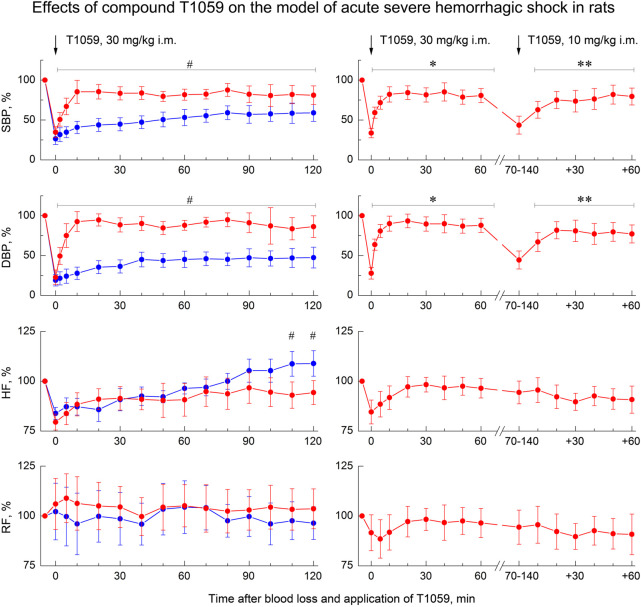
Effect of compound T1059 with a single i.m. injection at dose of 30 mg/kg (left) and two times i.m. injection at doses 30 + 10 mg/kg (right) on hemodynamics and external respiration of Wistar rats at an early stage of acute severe hemorrhagic shock. Red symbols and lines are indicators of T1059-treated rats (*n* = 12–16 per point), blue symbols and lines are indicators of untreated rats (*n* = 10–28). Data are normalized to the original values of indicators of animals and presented as percentage. Graphical deviations correspond to SD. # - significantly different indicators in untreated vs. T1059-treated rats (SBP: *p* = 0.04754, *p* = 0.01182, *p* = 0.00736, *p* = 0.00598, *p* = 0.00721, *p* = 0.00907, *p* = 0.00984, *p* = 0.01070, *p* = 0.00833, *p* = 0.00645, *p* = 0.02651, *p* = 0.03076, *p* = 0.04182, *p* = 0.03287, respectively; DBP: *p* = 0.02744, *p* = 0.00056, *p* = 0.00003, *p* < 0.00001, *p* = 0.00012, *p* = 0.00290, *p* = 0.00437, *p* = 0.00305, *p* = 0.00184, *p* = 0.00076, *p* = 0.00742, *p* = 0,01539, *p* = 0.01163, *p* = 0.00906, respectively; HF: *p* = 0.02747, *p* = 0.03825, respectively). * - significantly different with the indicator immediately after blood loss (SBP: *p* = 0.00817, *p* = 0.00543, *p* = 0.00072, *p* = 0.00061, *p* = 0.00085, *p* = 0.00069, *p* = 0.00107, *p* = 0.00084, respectively; DBP: *p* = 0.00023, *p* = 0.00014, *p* = 0.00007, *p* < 0.00001, *p* = 0.00036, *p* = 0.00009, *p* = 0.00031, *p* = 0.00023, respectively). **—significantly different with the indicator before the second injection of T1059 (SBP: *p* = 0.03850, *p* = 0.02725, *p* = 0.03468, *p* = 0.02954, *p* = 0.01309, *p* = 0.02083, respectively; DBP: *p* = 0.03941, *p* = 0.00807, *p* = 0.01625, *p* = 0.02736, *p* = 0.02012, *p* = 0.02547, respectively).

A single i. m. injection of T1059 at a dose of 30 mg/kg (1/9 LD10) also caused a pronounced vasopressor effect (Figure 4, left). In this case, T1059 hypertensive effect developed less rapidly - it increased monotonously in the first 10 min after injection, and then remained stably pronounced throughout the entire observation period. During this time period, the mean SBP and DBP in rats in severe hemorrhagic shock were 95–105 and 65–75 mmHg (respectively, 75%–85% and 80%–90% of the level before BL). The duration of a significant vasopressor effect of T1059 exceeded 120 min. Moreover, repeated i. m. administration of T1059 at a low dose (10 mg/kg) during the period when the vascular effect of the first injection (30 mg/kg) was significantly weakened allowed to prolong the vasopressor effect for at least another 60 min (Figure 4, right).

**FIGURE 5:**
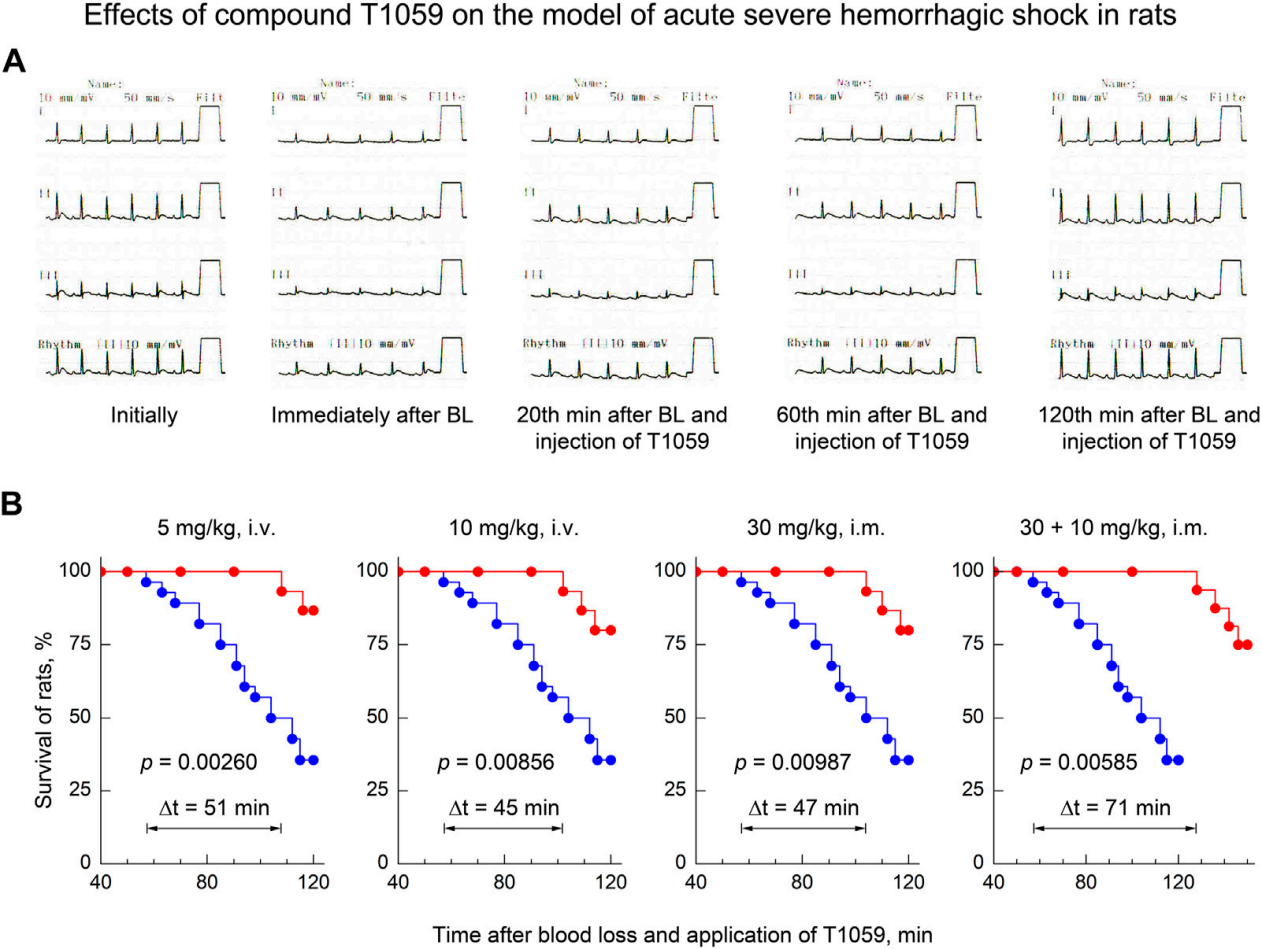
Effect of compound T1059 on the clinical course of the early stage of acute severe hemorrhagic shock in rats. **(A)** Typical cardio-respiratory dynamics in T1059-treated rats. Initially: BP—128/84 mmHg; HF—424 min-1; sinus rhythm, no changes in the ECG; RF—58 min-1. Immediately after blood loss (BL): BP—53/27; HF—335; sinus rhythm, R-wave depression; RF—63. 20 min after BL and injection of T1059: BP—95/66; HF—372; sinus rhythm, easing of R-wave depression, RF—60. 60 min after BL and injection of T1059: BP—120/81; HF—368; sinus rhythm, no changes in the ECG; RF—62. 120 min after BL and injection of T1059: BP—115/74; HF—404; sinus rhythm, moderate sings of myocardial ischemia; RF—59. **(B)** Effect of compound T1059 at different doses and applications on the short-term survival of rats. Red symbols and curves—survival of T1059-treated rats (*n* = 15–16), blue symbols and curve—survival of untreated rats (*n* = 28). Diagrams were plotted using Kaplan-Meier method. *p*—significance level of the difference in the survival of T1059-treated vs. untreated rats; Δt—an increase in time of the non-lethal course of shock when using T1059.

Moreover, along with the manifestations of a pronounced stable vasopressor activity of T1059, its significant influence on the clinical course of the early stage of severe acute hemorrhagic shock attracted attention. As noted above, in most control rats in this model, cardiorespiratory insufficiency increased 40–70 min after BL. The death of a significant part (64%) of these animals was recorded at the 2^nd^ hour of observation (Figure 2). At the same time, long-term stabilization of the hemodynamics of T1059-treated animals at all doses and methods of administration significantly “restrained” the development of threatening complications. Monitoring data indicated the increase in significant cardio-respiratory disorders only in 20%–30% of T1059-treated rats and at later periods (90–110 min after BL and T1059 administration). In most of these animals, negative symptoms were limited to mild signs of myocardial ischemia at the end of monitoring (Figure 5A). Moreover, a positive objective cardio-respiratory dynamics was accompanied by a statistically significant increase in the short-term survival of T1059-treated rats (Figure 5B)—by 120–150 min of observation, lethality in these groups of animals was 13%–25%. Comparison of survival diagrams showed a significant (1.8–2.3 times) increase in the time of non-lethal shock in T1059-treated groups—from 57 min in control rats to 102–108 min in T1059-treated rats with a single injection (5 and 10 mg/kg, i.v. or 30 mg/kg, i m.) and to 128 min with 2-fold injection (30 + 10 mg/kg, i.m.).

### Effects of compound T1059 on the model of acute hemorrhagic shock in dogs

**FIGURE 6:**
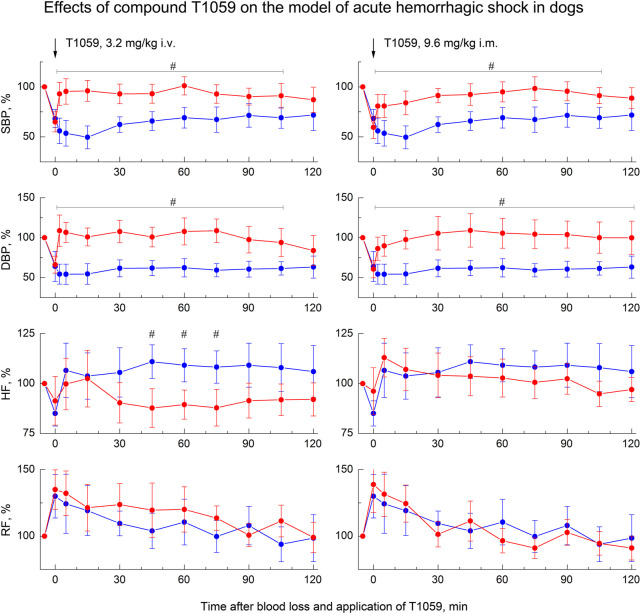
Effect of compound T1059 with a single i.v. injection at dose of 3.2 mg/kg (left) and a single i.m. injection at dose 9.6 mg/kg (right) on hemodynamics and external respiration of dogs at an early stage of acute hemorrhagic shock. Red symbols and lines are indicators of T1059-treated dogs (*n* = 8), blue symbols and lines are indicators of untreated dogs (*n* = 7). Data are normalized to the original values of indicators of animals and presented as percentage. Graphical deviations correspond to SD. #—significantly different indicators in untreated vs. T1059-treated dogs (3.2 mg/kg i.v, SBP: *p* = 0.00478, *p* = 0.00253, *p* = 0.00069, *p* = 0.00625, *p* = 0.00814, *p* = 0.00167, *p* = 0.01350, *p* = 0.03902, *p* = 0.02733, respectively. DBP: *p* = 0.00017, *p* = 0.00009, *p* = 0.00034, *p* = 0.00086, *p* = 0.00295, *p* = 0.00071, *p* = 0.00053, *p* = 0.00492, *p* = 0.01768, respectively. HF: *p* = 0.00745, *p* = 0.00908, *p* = 0.01362, respectively. 9.6 mg/kg i.m, SBP: *p* = 0.02176, *p* = 0.01892, *p* = 0.00354, *p* = 0.00513, *p* = 0.00940, *p* = 0.00874, *p* = 0.00721, *p* = 0.01956, *p* = 0.02498, respectively. DBP: *p* = 0.01572, *p* = 0.00743, *p* = 0.00209, *p* = 0.00651, *p* = 0.00375, *p* = 0.00624, *p* = 0.00176, *p* = 0.00507, *p* = 0.00943, *p* = 0.02745, respectively).

A less severe model of acute hemorrhagic shock was used to study the vascular and hemodynamic effects of T1059 in dogs. In this case, a less acute (within 20–25 min) controlled loss of 25% of circulating blood led to the development of moderately severe hemorrhagic shock in animals (Figure 6). 2–5 min after the end of BL, SBP/DBP in dogs averaged 65/45 mmHg (52–55% of the level before BL). In the first 30–40 min after BL, a slight compensation of hypotension due to a moderate acceleration of the heart rate and, apparently, an increase in stroke volume was registered in control, untreated animals. And further until the end of the observation, the SBP/DBP indicators in these animals remained at the level of 75/45 mmHg (respectively, 65% and 55% of the level before BL). Further, during 120 min of the experiment (and in the next 15 days) no any death or threatening clinical symptoms and ECG changes in the control untreated group were registered.

The vasopressor activity of T1059 was studied in this model with a single parenteral administration at doses of 3.2 mg/kg (i.v.) and 9.5 mg/kg (i.m.). That is equivalent to doses of 10 and 30 mg/kg for rats, taking into account the species surface area of the animal’s body (Mironov, 2012). Dogs of the experimental groups endured slow i. v. and i.m. injections of T1059 solutions easily and painlessly. No any manifestations of intoxication or changes in the state and behavior of these animals were observed. Subsequent examinations also showed no any clinical manifestations of local damaging effects of T1059.

At the same time, T1059 in this model caused almost the same vascular and hemodynamic effects in hypotensive dogs as in hypotensive rats. After a single i.v. injection of T1059, 3.2 mg/kg, a stably pronounced hypertensive effect was observed during the first 60–75 min, it was achieved already at the 5^th^ minute after the injection (Figure 5, left). During this time period, SBP and DBP in dogs were 110–125 and 80–90 mmHg (90%–100% and 100–110% of pre-BL levels). In the next 20–30 min, the effect manifestation decreased slightly - the average values of SBP and DBP were 105 and 75 mmHg (85% and 90% of pre-BL levels). The total duration of T1059 significant vasopressor effect was 115 min. After a single i.m. injection of T1059, 9.5 mg/kg, a significant effect was also observed as early as 5 min after injection (Figure 5, right). During the first 45–60 min, its manifestation increased from 100/70 mmHg (80% and 85% of pre-BL level) to 120/90 mmHg (95% and 105% of pre-BL level). The total duration of T1059 significant vasopressor effect in this case was also 115 min, and increased DBP values were observed until the end of the experiment.

## Discussion

Since the discovery of the NOS/sGC/cGMP pathway of vascular relaxation in the 1980’s (Furchgott and Zawadzki, 1980; Palmer et al., 1987; Rees et al., 1989), our understanding of the physiological and pathophysiological role of NO and various NOS isoforms in regulation of vascular tone and hemodynamics are constantly refined and expanded. Nevertheless, since that time, the vasoconstrictive, vasopressor ability of effective eNOS and iNOS inhibitors blocking the vascular relaxation pathway has been perceived rather as a natural property. Indeed, to date, such an activity has been shown for many NOS inhibitors containing guanidine (L-NMMA, L-NNA, L-NAME), aminoguanidine (Kilbourn et al., 1990, 1997; Wu et al., 1995; Avontuur et al., 1998), amidine (L-NIL, 7-NI, 1400 W) (Faraci and Brian, 1995; Wray et al., 1998; Kadoi and Goto, 2007) and thioamidine group (derivatives of isothiourea, L-thiocytrulline, 2-aminothiazole/thiazoline/thiazine) (Fray et al., 1994; Narayanan et al., 1995; Handy et al., 1996; Proskuryakov et al., 2002, 2004, 2010; Filimonova et al., 2012b; Nurieva et al., 2018; Alexeev et al., 2019). Apparently, the isothiourea derivative T1059 is not an exception in this series.

According to radiological studies on isolated NOS isoforms (Filimonova et al., 2018), compound T1059 is a fairly effective inhibitor of eNOS and iNOS (its IC_50_ values are 3.3 and 1.8 μM, respectively). It not inferior in such activity to L-NMMA and L-NNA (Faraci et al., 1996; Boer et al., 2000). Apparently, the features of N-substituting radical in chemical structure of this isothiourea make it difficult to interact its thioamidine fragment with nNOS active site (its IC_50_ value is 60.1 μM). This provides T1059 a significant (15–30 times) selectivity for eNOS and iNOS,—NOS isoforms that play a leading role in the physiological and pathophysiological regulation of vascular homeostasis and, in particular, vascular tone (Napoli and Ignarro, 2009; Forstermann and Sessa, 2012; Krol and Kapinska, 2021).

Such features of T1059 biochemical activity probably determine the presence of a pronounced vasopressor activity in this compound. In our studies on models of acute hemorrhagic shock, T1059, after a single parenteral administration in sufficiently safe doses (1/50–1/9 LD_10_), caused in rats and dogs a rapid increase in vascular tone, accompanied by a prolonged hypertensive effect (within 90–120 min in rats, and within 115 min in dogs). The repeated administration of T1059 at low doses (1/3 of the dose of the first injection) made it possible to considerably (by at least 60 min) prolong a significant vasopressor effect.

Routine use of vasopressors is currently not recommended in the treatment of acute posthemorrhagic hypotension because of possible excessive centralization of blood flow, that can accelerate the development of multiple organ failure and aggravate « delayed » damage during reperfusion. Options for fluid resuscitation are considered as optimal strategies. However, fluid replacement in such disorders may also be unsafe and ineffective (e.g, in uncontrolled traumatic hemorrhagic shock or in states resistant to volume therapy) or belated. So, with the loss of the “golden hour,” the chances of survival of patients with acute severe hemorrhagic shock in the preclinical setting remain low. In this regard, a significant positive effect of T1059 on the clinical course of the early stage of acute severe hemorrhagic shock in rats seems to be an important result of this study. In all schemes, T1059 administration considerably inhibited the development of threatening cardiorespiratory disorders and significantly (*p* = 0.0026–0.0098) increased the short-term survival of experimental animals, formally extending the duration of the “golden hour” by 2 times.

This “therapeutic” effect of T1059 in this case, most likely, is realized due to a fairly long stabilization of animals’ hemodynamics. It reproduces the positive effects of vasopressor support in the early stages of severe shock, that have been shown in a number of experimental works for adrenomimetics and peptide vasoconstrictors (Hakstian et al., 1961; Nouira et al., 2005; Johnson et al., 2006; Sanui et al., 2006; Poloujadoff et al., 2007; Beloncle et al., 2013; Djogovic et al., 2015). At the same time, the contribution of the specific NOS inhibitory activity of T1059 to this positive effect cannot be excluded. So, a number of studies have shown the ability of some NOS inhibitors to increase short-term survival in the early stages of severe hemorrhagic shock. Such an effect can be exerted not only by NOS inhibitors with high vasopressor activity (Khazaei et al., 2012), but also by selective iNOS inhibitors (McDonald et al., 2002; Soliman, 2014). Therefore, the nature of T1059 NOS-inhibitory activity is capable of providing not only to implement vasopressor stabilization of hemodynamics in severe hemorrhagic shock, but also to limit the development of destructive processes caused by oxidative and nitrosative stresses against the background of acute hypoxia.

## Conclusion

Studies on models of acute hemorrhagic hypotension in rats and dogs demonstrate that the NOS inhibitor T1059 is a potent long-acting vasopressor. It caused the rapid development of a stable and prolonged (within 90–120 min) hypertensive effect after a single parenteral administration in sufficiently safe doses (1/50–1/9 LD_10_) in hypotensive animals. The repeated administration of T1059 at low doses made it possible to prolong the vasopressor effect. Long-term stabilization of hemodynamics in T1059-treated rats at an early stage of severe hemorrhagic shock significantly increased the short-term survival of animals. These data suggest that NOS inhibitors and, in particular, compound T1059, are able to create new opportunities in the treatment of hypotensive disorders, including the provision of assistance at the prehospital stage of treatment of such pathologies.

## Data Availability

The raw data supporting the conclusions of this article will be made available by the authors, without undue reservation.

## Abbreviations

NO, nitric oxide; NOS, nitric oxide synthase; BL, blood loss; BP, blood pressure; SBP and DBP, systolic and diastolic BP; HF, heart frequency; HR, respiratory frequency.

## References

[B1] Alexeev A. A. Nurieva E. V. Trofimova T. P. Chesnakova E. A. Grishin Y. K. Lyssenko K. A. (2019). Bicyclic bridged isothioureas: Synthesis and evaluation of activity in a model of lipopolysaccharide-induced septic shock. Mendeleev Commun. 29, 14–16. 10.1016/j.mencom.2019.01.003

[B2] Avontuur J. A. Tutein Nolthenius R. P. van Bodegom J. W. Bruining H. A. (1998). Prolonged inhibition of nitric oxide synthesis in severe septic shock: A clinical study. Crit. Care Med. 26, 660–667. 10.1097/00003246-199804000-00012 9559602

[B3] Beloncle F. Meziani F. Lerolle N. Radermacher P. Asfar P. (2013). Does vasopressor therapy have an indication in hemorrhagic shoch? Ann. Intensive Care 3, 13. 10.1186/2110-5820-3-13 23697682 PMC3691630

[B4] Berezovskaya I. V. (2003). Classification of substances with respect to acute toxicity for parenteral administration. Pharm. Chem. J. 37, 139–141. 10.1023/A:1024586630954

[B5] Boer R. Ulrich W. R. Klein T. Mirau B. Haas S. Baur I. (2000). The inhibitory potency and selectivity of arginine substrate site nitric-oxide synthase inhibitors is solely determined by their affinity toward the different isoenzymes. Mol. Pharmacol. 58, 1026–1034. 10.1124/mol.58.5.1026 11040050

[B6] Diehl K. H. Hull R. Morton D. Pfister R. Rabemampianina Y. Smith D. (2001). A good practice guide to the administration of substances and removal of blood, including routes and volumes. J. Appl. Toxicol. 21, 15–23. 10.1002/jat.727 11180276

[B7] Djogovic D. MacDonald S. Wensel A. Green R. Loubani O. Archambault P. (2015). Vasopressor and inotrope use in Canadian Emergency Departments: Evidence based consensus guidelines. Can. J. Emerg. Med. 17, 1–16. 10.1017/cem.2014.77 25961083

[B8] Faraci F. M. Brian J. E. Jr (1995). 7-Nitroindazole inhibits brain nitric oxide synthase and cerebral vasodilatation in response to N-methyl-d-aspartate. Stroke 26, 2172–2175. 10.1161/01.str.26.11.2172 7482668

[B9] Faraci W. S. Nagel A. A. Verdries K. A. Vincent L. A. Xu H. Nichols L. E. (1996). 2-amino-4-methylpyridine as a potent inhibitor of inducible NO synthase activity *in vitro* and *in vivo* . Br. J. Pharmacol. 119, 1101–1108. 10.1111/j.1476-5381.1996.tb16010.x 8937711 PMC1915898

[B10] Filimonova M. Saburova A. Makarchuk V. Shevchenko L. Surinova V. Yuzhakov V. (2021). The ability of the nitric oxide synthases inhibitor T1023 to selectively protect the non-malignant tissues. Int. J. Mol. Sci. 22, 9340. 10.3390/ijms22179340 34502247 PMC8431509

[B11] Filimonova M. Saburova A. Shevchenko L. Makarchuk V. Shitova A. Soldatova O. (2022b). 1-Isobutanoil-2-isopropylisothiourea phosphate, T1082: A safe and effective prevention of radiotherapy complications in oncology. Int. J. Mol. Sci. 23, 2697. 10.3390/ijms23052697 35269835 PMC8911053

[B12] Filimonova M. Shitova A. Soldatova O. Shevchenko L. Saburova A. Podosinnikova T. (2022a). Combination of NOS-and PDK-inhibitory activity: Possible way to enhance antitumor effects. Int. J. Mol. Sci. 23, 730. 10.3390/ijms23020730 35054914 PMC8775993

[B13] Filimonova M. V. Makarchuk V. M. Shevchenko L. I. Saburova A. S. Surinova V. I. Izmestieva O. S. (2020b). Radioprotective activity of the nitric oxide synthase inhibitor T1023. Toxicological and biochemical properties, cardiovascular and radioprotective effects. Radiat. Res. 194, 532–543. 10.1667/RADE-20-00046.1 34609510

[B14] Filimonova M. V. Podosinnikova T. S. Samsonova A. S. Makarchuk V. M. Shevchenko L. I. Filimonov A. S. (2019b). Comparison of antitumor effects of combined and separate treatment with NO synthase inhibitor T1023 and PDK1 inhibitor dichloroacetate. Bull. Exp. Biol. Med. 168, 92–94. 10.1007/s10517-019-04655-1 31768777

[B15] Filimonova M. V. Proskuriakov S. I. Shevchenko L. I. Shevchuk A. S. Lushnikova G. A. Makarchuk V. M. (2012a). [Radioprotective properties of isothiourea derivatives with NO-inhibitory mechanism of action]. Radiats. Biol. Radioecol. 52, 593–601. (In Russian). 23516890

[B16] Filimonova M. V. Shevchenko L. I. Makarchuk V. M. Chesnakova E. A. Shevchuk A. S. Filimonov A. S. (2020a). Vasopressor properties of NO synthase inhibitor T1059. Part II. Hemodynamic effects on hypovolemic disorders. Pharm. Chem. J. 53, 1113–1117. 10.1007/s11094-020-02132-y

[B17] Filimonova M. V. Shevchenko L. I. Makarchuk V. M. Chesnakova E. A. Surinova V. I. Shevchuk A. S. (2018). Vasopressor properties of nitric oxide synthase inhibitor T1059. Part I: Synthesis, toxicity, NOS-inhibition activity, and hemodynamic effects under normotensive conditions. Pharm. Chem. J. 52, 294–298. 10.1007/s11094-018-1809-2

[B18] Filimonova M. V. Shevchenko L. I. Makarchuk V. M. Chesnakova E. A. Tsyb A. F. (2015). Inventor; medical radiological research center of the Ministry of health of the Russian federation, assignee. Vasopressor agent. Russ. Fed. Pat. RU 2, 552–529.

[B19] Filimonova M. V. Trofimova T. P. Borisova G. S. Mandrugin A. A. (2012b). Antihypotensive activity of 2-acetylamino-5, 6-dihydro-4H-1, 3-thiazine for an endotoxic shock model in rats. Pharm. Chem. J. 46, 210–212. 10.1007/s11094-012-0763-7

[B20] Filimonova M. V. Yuzhakov V. V. Filimonov A. S. Makarchuk V. M. Bandurko L. N. Korneeva T. S. (2019a). Comparative study of the effects of NOS inhibitor T1023 and bevacizumabum on growth and morphology of Lewis lung carcinoma]. Patologicheskaya Fiziologiya i Eksperimentalnaya Terapiya = Pathological Physiology Exp. Ther. 63, 89–98. (In Russian). 10.25557/0031-2991.2019.02.89-98

[B21] Forstermann U. Sessa W. C. (2012). Nitric oxide synthases: Regulation and function. Eur. Heart J. 33, 829–837. 10.1093/eurheartj/ehr304 21890489 PMC3345541

[B22] Fray C. Narayanan K. McMillan K. Spack L. Gross S. S. Masters B. S. (1994). L-thiocitrulline. A stereospecific, heme-binding inhibitor of nitric-oxide synthases. J. Biol. Chem. 269, 26083–26091. 10.1016/S0021-9258(18)47162-1 7523401

[B23] Furchgott R. F. Zawadzki J. V. (1980). The obligatory role of endothelial cells in the relaxation of arterial smooth muscle by acetylcholine. Nature 288, 373–376. 10.1038/288373a0 6253831

[B24] Gad S. G. (Editor) (2007). Animal models in toxicology. 2nd Edition (New York: Taylor & Francis), 952.

[B25] Hakstian R. W. Hampson L. G. Gurd F. N. (1961). Pharmacological agents in experimental hemorrhagic shock. A controlled comparison of treatment with hydralazine, hydrocortisone, and levarterenol (1-norepinephrine). Arch. Surg. 3, 335–347. 10.1001/archsurg.1961.01300150009002 13710650

[B26] Handy R. L. Wallace P. Moore P. K. (1996). Inhibition of nitric oxide synthase by isothioureas: Cardiovascular and antinociceptive effects. Pharmacol. Biochem. Behav. 55, 179–184. 10.1016/s0091-3057(96)00051-2 8951952

[B27] Hogg N. (2010). Detection of nitric oxide by electron paramagnetic resonance spectroscopy. Free Radic. Biol. Med. 49, 122–129. 10.1016/j.freeradbiomed.2010.03.009 20304044 PMC2916063

[B28] Holm S. (1979). A simple sequentially rejective multiple test procedure. Scand. J. Statistics 6, 65–70.

[B29] Johnson K. B. Pearce F. J. Jeffreys N. McJames S. W. Cluff M. (2006). Impact of vasopressin on hemodynamic and metabolic function in the decompensatory phase of hemorrhagic shock. J. Cardiothorac. Vasc. Anesth. 3, 167–172. 10.1053/j.jvca.2005.11.015 16616655

[B30] Kadoi Y. Goto F. (2007). Effects of selective iNOS inhibition on systemic hemodynamics and mortality rate on endotoxic shock in streptozotocin-induced diabetic rats. Shock 28, 602–609. 10.1097/SHK.0b013e31804d452d 17607161

[B31] Khazaei M. Barmaki B. Nasimi A. (2012). Protective role of selective nitric oxide synthase inhibitor for treatment of decompensated hemorrhagic shock in normotensive and hypertensive rats. Int. J. Prev. Med. 3, 47–53. PMID: 22355477 PMCID: PMC3278869. 22355477 PMC3278869

[B32] Kilbourn R. G. Jubran A. Gross S. S. Griffith O. W. Levi R. Adams J. (1990). Reversal of endotoxin-mediated shock by NG-methyl-L-arginine, an inhibitor of nitric oxide synthesis. Biochem. Biophys. Res. Commun. 172, 1132–1138. 10.1016/0006-291x(90)91565-a 2244897

[B33] Kilbourn R. G. Szab C. Trauber D. L. (1997). Beneficial versus detrimental effects of nitric oxide synthase inhibitors in circulatory shock: Lessons learned from experimental and clinical studies. Shock 7, 235–246. 10.1097/00024382-199704000-00001 9110408

[B34] Krol M. Kapinska M. (2021). Human nitric oxide synthase-its functions, polymorphisms, and inhibitors in the context of inflammation, diabetes and cardiovascular diseases. Int. J. Mol. Sci. 22, 56. 10.3390/ijms22010056 PMC7793075 33374571

[B35] Lim R. K. Rink K. G. Glass H. G. Soaje-Echague E. (1961). A method for the evaluation of cumulation and tolerance by the determination of acute and subchronic median effective doses. Arch. Int. Pharmacodyn. Ther. 130, 336–353. PMID: 13762162. 13762162

[B36] McDonald M. C. Izumi M. Cuzzocrea S. Thiemermann C. (2002). A novel, potent and selective inhibitor of the activity of inducible nitric oxide synthase (GW274150) reduces the organ injury in hemorrhagic shock. J. Physiol. Pharmacol. 53, 555–569. PMID: 12512692. 12512692

[B37] Mironov A. N. (Editor) (2012). Guidelines for preclinical Drug research (Moscow, Russia: Grif & Co.), 944. (In Russian). Part

[B38] Napoli C. Ignarro L. J. (2009). Nitric oxide and pathogenic mechanisms involved in the development of vascular diseases. Arch. Pharm. Res. 32, 1103–1108. 10.1007/s12272-009-1801-1 19727602

[B39] Narayanan K. Spack L. McMillan K. Kilbourn R. G. Hayward M. A. Masters B. S. (1995). S-alkyl-L-thiocitrullines. Potent stereoselective inhibitors of nitric oxide synthase with strong pressor activity *in vivo* . J. Biol. Chem. 270, 11103–11110. 10.1074/jbc.270.19.11103 7538112

[B40] Nouira S. Elatrous S. Dimassi S. Besbes L. Boukef R. Mohamed B. (2005). Effects of norepinephrine on static and dynamic preload indicators in experimental hemorrhagic shock. Crit. Care Med. 3, 2339–2343. 10.1097/01.CCM.0000182801.48137.13 16215390

[B41] Nurieva E. V. Trofimova T. P. Alexeev A. A. Proshin A. N. Chesnakova E. A. Grishin Y. K. (2018). Synthesis and antihypotensive properties of 2-amino-2-thiazoline analogues with enhanced lipophilicity. Mendeleev Commun. 28, 390–392. 10.1016/j.mencom.2018.07.016

[B42] Palmer R. M. J. Ferrige A. G. Moncada S. (1987). Nitric oxide release accounts for the biological activity of endothelium-derived relaxing factor. Nature 327, 524–526. 10.1038/327524a0 3495737

[B43] Poloujadoff M. P. Borron S. W. Amathieu R. Favret F. Camara M. S. Lapostolle F. (2007). Improved survival after resuscitation with norepinephrine in a murine model of uncontrolled hemorrhagic shock. Anesthesiology 3, 591–596. 10.1097/01.anes.0000281926.54940.6a 17893455

[B44] Proskuryakov S. Y. Filimonova M. V. Borovaya O. N. Kucherenko N. G. Trishkina A. I. Steyn L. V. (2010). Effect of NO inhibitors on hypovolemic shock-induced hypotension. Bull. Exp. Biol. Med. 150, 18–22. 10.1007/s10517-010-1057-2 21161041

[B45] Proskuryakov S. Y. Filimonova M. V. Verkhovskii Y. G. Konoplyannikov A. G. Mandrugin A. A. Fedoseev V. M. (2004). Effect of NO synthase inhibitor 2-amino-5, 6-dihydro-4H-1, 3-thiazine on endotoxin-induced changes in hemodynamic parameters and respiration in rats. Bull. Exp. Biol. Med. 138, 397–400. 10.1007/s10517-005-0110-z 15665955

[B46] Proskuryakov S. Y. Konoplyannikov A. G. Skvortzov V. G. Mandrugin A. A. Fedoseev V. M. (2005). Nitric oxide synthase inhibitors containing the carboxamidine group or its isosteres. Russ. Chem. Rev. 74, 859–870. 10.1070/RC2005v074n09ABEH000923

[B47] Proskuryakov S. Y. Kucherenko N. G. Trishkina A. I. Filimonova M. V. Shevchuk A. G. Shtein L. V. (2002). NO-inhibiting and vasotropic activity of some compounds with thioamidine group. Bull. Exp. Biol. Med. 134, 338–341. 10.1023/A:1021943811672 12533753

[B48] Rees D. D. Palmer R. M. J. Moncada S. (1989). Role of endothelium-derived nitric oxide in the regulation of blood pressure. Proc. Natl. Acad. Sci. U. S. A. 86, 3375–3378. 10.1073/pnas.86.9.3375 2497467 PMC287135

[B49] Saburova A. S. Filimonova M. V. Yuzhakov V. V. Shevchenko L. I. Yakovleva N. D. Bandurko L. N. (2020). The influence of nitric oxide synthases inhibitor Т1023 on the development of radiation pneumofibrosis in rats. Radiatsionnaya Gygiena = Radiat. Hyg. 13, 60–67. (In Russian). 10.21514/1998-426X-2020-13-1-60-67

[B50] Sanui M. King D. R. Feinstein A. J. Varon A. J. Cohn S. M. Proctor K. G. (2006). Effects of arginine vasopressin during resuscitation from hemorrhagic hypotension after traumatic brain injury. Crit. Care Med. 3, 433–438. 10.1097/01.CCM.0000196206.83534.39 16424725

[B51] Soliman M. M. (2014). Effects of aminoguanidine, a potent nitric oxide synthase inhibitor, on myocardial and organ structure in a rat model of hemorrhagic shock. J. Emerg. Trauma Shock 7, 190–195. 10.4103/0974-2700.136864 25114430 PMC4126120

[B52] van Eijk H. M. Luiking Y. C. Deutz N. E. (2007). Methods using stable isotopes to measure nitric oxide (NO) synthesis in the L-arginine/NO pathway in health and disease. J. Chromatogr. B Anal. Technol. Biomed. Life Sci. 851, 172–185. 10.1016/j.jchromb.2006.08.054 17049318

[B53] Wray G. M. Millar C. G. Hinds C. J. Thiemermann C. (1998). Selective inhibition of the activity of inducible nitric oxide synthase prevents the circulatory failure, but not the organ injury/dysfunction, caused by endotoxin. Shock 9, 329–335. 10.1097/00024382-199805000-00003 9617881

[B54] Wu C. C. Chen S. J. Szabo C. Thiemermann C. Vane J. R. (1995). Aminoguanidine attenuates the delayed circulatory failure and improves survival in rodent models of endotoxic shock. Br. J. Pharmacol. 114, 1666–1672. 10.1111/j.1476-5381.1995.tb14955.x 7541282 PMC1510405

